# More than half of data deficient species predicted to be threatened by extinction

**DOI:** 10.1038/s42003-022-03638-9

**Published:** 2022-08-04

**Authors:** Jan Borgelt, Martin Dorber, Marthe Alnes Høiberg, Francesca Verones

**Affiliations:** Industrial Ecology Programme, Department of Energy and Process Engineering, Norwegian University of Science and Technology (NTNU), Trondheim, Norway

**Keywords:** Biodiversity, Ecological modelling, Machine learning, Conservation biology

## Abstract

The IUCN Red List of Threatened Species is essential for practical and theoretical efforts to protect biodiversity. However, species classified as “Data Deficient” (DD) regularly mislead practitioners due to their uncertain extinction risk. Here we present machine learning-derived probabilities of being threatened by extinction for 7699 DD species, comprising 17% of the entire IUCN spatial datasets. Our predictions suggest that DD species as a group may in fact be more threatened than data-sufficient species. We found that 85% of DD amphibians are likely to be threatened by extinction, as well as more than half of DD species in many other taxonomic groups, such as mammals and reptiles. Consequently, our predictions indicate that, amongst others, the conservation relevance of biodiversity hotspots in South America may be boosted by up to 20% if DD species were acknowledged. The predicted probabilities for DD species are highly variable across taxa and regions, implying current Red List-derived indices and priorities may be biased.

## Abstract (web summary)

Data Deficient species are more likely to be at extinction risk than previously thought across multiple taxonomic groups.

## Introduction

Measuring ongoing and anticipating potential threats is vital for preventing damage to the natural world^1–8^, which entails detailed knowledge about the current state of biodiversity. A central data resource enabling a multitude of overarching analyses in conservation and sustainability science^9^ is the International Union for the Conservation of Nature (IUCN)’s Red List of Threatened Species (hereafter: Red List). The Red List assesses extinction risks and reports Red List categorization for more than 140,000 species based on a set of quantitative criteria^10^ relying for instance on extent of occurrence, area of occupancy, population trends, or population size. However, the sheer amount of known and unknown species globally^11,12^, the dynamic nature of threats and trends^7^, and limited human resources for undertaking such Red List assessments^13,14^ turn this critical endeavour into a Sisyphean task.

Consequently, only a small proportion of known species have been assessed for their conservation priority so far^15,16^, unevenly distributed across space, time and taxa^13,16^. In addition, numerous assessed species are classified as Data Deficient (DD) even in otherwise comprehensively assessed species groups. A species is considered DD if there is “inadequate information to make a direct, or indirect, assessment of its risk of extinction based on its distribution and/or population status”^17^. More specifically Bland et al. identified 8 main justifications as to why species are assessed as DD: uncertain provenance, type series, few records (<5), old records (before 1970), uncertain population status or distribution, uncertain threats, new species (discovered in the last 10 years), and taxonomic uncertainty^18^. In parallel, Butchart and Bird stated that the DD category “is probably the most controversial and misunderstood Red List category”^19^. One of the main reasons are value choices when dealing with uncertainty and applying the IUCN Guidelines. If, due to uncertain data, a species can be listed as Critically Endangered (CR) and Least Concern (LC), the species should be listed as DD. However, if the assessor considers a species being not LC but is unsure about its exact threat-level, DD is not the appropriate category. In this case, the assessor needs to decide and assign the species to a category, i.e., risk tolerance. It is important to note that we do not distinguish the DD species according to the reason for their classification as DD^17^.

On average across all taxa and regions, one of six assessed species is classified as DD^15,18,20^. Although DD species are sometimes treated as being not threatened^21^, studies suggest that they are of particular conservation importance because a higher portion of them may be threatened by extinction compared to data-sufficient (DS) species^22–24^. However, since DD species could belong to any Red List category, they are difficult to handle for practitioners^21,25^ and are therefore generally ignored in studies analysing biodiversity impacts and change^26,27^. For instance, the Red List Index^27^ is built upon well-assessed threat-levels for individual species at several points in time and directly applied in, e.g., sustainable development goals^28^ and biodiversity targets^29^. In addition, studies linking biodiversity loss to global trade footprints^30,31^ and approaches to transform threat-levels to numerical conservation indicators^32^ have ignored DD species. Similarly, the recently suggested metric^26^ for measuring success of the post-2020 Global Biodiversity Framework will not be applicable for DD species.

In stark contrast, the continuous growth in knowledge turnover during the digital era has resulted in constant improvement in the availability of global data on biodiversity, human activities, and environmental threats^33^. Statistical tools, such as machine learning (ML), can detect relevant signals in large datasets, thereby offering a time- and cost-effective approach to tackle data deficiency^34–37^. The utility of ML models for predicting species’ extinction risk or conservation status was successfully proven for species in single taxonomic groups with great accuracy^24,38–44^, regionally as well as globally. However, such predictions are needed consistently for all relevant species to effectively benefit global conservation and sustainability analyses^16^.

Here, we present a global multitaxon ML classifier that predicts the probability of being threatened by extinction (hereafter: PE score) based on, amongst others, species taxonomy, range extent, and summarized stressors (min., max., mean and median) within species range maps, as well as species occurrence cells (0.5-degree cells). The classifier was trained and tested on threat levels for 28,363 DS species, drawing on selected features out of more than 400 predictors, human pressures, and environmental stressors. We applied the classifier to predict PE scores for DD species (*n* = 7699) that include range maps of their distribution in their IUCN Red List database record (Version 2020-3)^45,46^, to our knowledge the largest data provider of range maps for thousands of species. Since biodiversity varies greatly through space, it is crucial to perform assessments in a spatially explicit way and include their entire spatial extent.

## Results and discussion

### Classifier performance

**Fig. 1:**
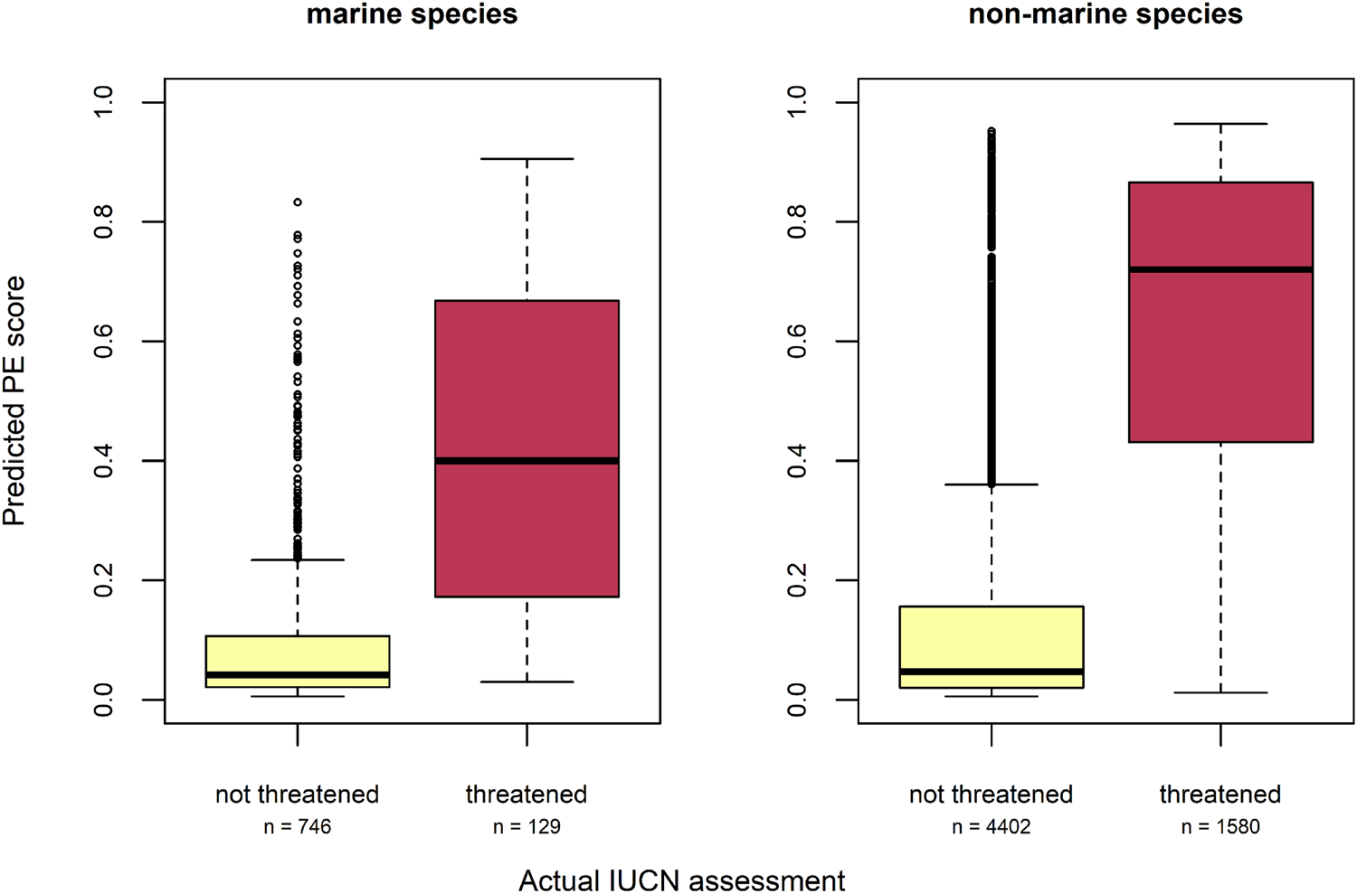
Predicted scores for threatened versus not threatened species. Boxplot showing the interquartile range (box), median (black line), minimum and maximum values without outliers (error bars), and outliers (points) of predicted probability of being threatened by extinction (PE score) across the actual IUCN assessment (not threatened and threatened) for marine (*n* = 875) and non-marine (*n* = 5982) species in the set-aside testing data.

The trained classifier was able to successfully separate between threatened and non-threatened species within a set-aside testing dataset, as well as continuous predictions (i.e., PE scores) (Fig. 1). The binary classifier obtained an overall accuracy of 85% (Table 1), being more precise in predicting which species are not threatened by extinction than in predicting which species are threatened. 93% and 92% of species that we predicted to be not threatened were indeed not threatened (for marine and non-marine species respectively). Hence, with only 7–8% of negative predictions (i.e., predicted as not threatened) being incorrect, we are confident that our binary classifier avoids underestimating the conservation status of most taxa. Instead, the binary classifier may be prone to overestimating the status of some taxa; only 60% to 67% of species that we predicted to be threatened are also classified as threatened by the IUCN (for marine and non-marine species respectively). The continuous classifier, however, seems to only underestimate the risk for marine species when directly compared to non-marine species. The relative ranking of continuous predictions within the groups remains valid for all species (AUC = 0.91, AUC_PR_ = 0.80, Gini-Coefficient = 0.82) and across taxonomic classes (Supplementary Table 1). Hence, on average, species being threatened by extinction obtain higher predicted PE scores than not threatened species, for both marine and non-marine species (Fig. 1). Binary as well as continuous predictions across marine versus non-marine groups perform well but are not directly comparable.

**Table 1:** Classifier performance.

	Reference			
Predicted	Not threatened	Threatened	Not threatened	Threatened
Not threatened	695 (26)	54 (10)	3786 (20)	309 (4)
Threatened	51 (8)	75 (25)	616 (7)	1271 (23)

	Marine species	Non-marine species
Accuracy	0.88 (0.74)	0.85 (0.80)
Specificity	0.93 (0.76)	0.86 (0.74)
Sensitivity	0.58 (0.71)	0.80 (0.85)
Negative Pred. Value	0.93 (0.72)	0.92 (0.83)
Positive Pred. Value	0.60 (0.76)	0.67 (0.77)
Balanced Accuracy	0.76 (0.74)	0.83 (0.80)

Confusion matrix and resulting performance measures for marine and non-marine species based on the set-aside testing data (25% of the dataset) and based on formerly Data Deficient species (*n* = 123) in IUCN version 2021-2 (in brackets).

We further tested our classifier against an IUCN update (Version 2021-2)^15^ that was released after our model was trained (Supplementary Fig. 1). In this update, we found that 123 former DD species from Version 2020-3 were now assigned a threat-level. Our classifier labelled 94 of those species (76%) correctly (Table 1), being equally precise in predicting whether the species was threatened (76%) or not threatened (77%) but more accurate for non-marine (80%) than for marine species (74%).

### Data deficient species are more threatened by extinction than data-sufficient species

On average we obtained higher PE scores for DD species (43%) than for DS species (26%), resulting in 56% of DD species (*n* = 4336) predicted to be threatened by extinction (Supplementary Table 1) versus 28% of DS species^46^. The generated predictions reinforce the concern that DD species are of high conservation interest^21,25^ and, given the large variance in predicted probabilities of being threatened (Supplementary Fig. 2), highlight the importance of treating DD species individually.

**Fig. 2:**
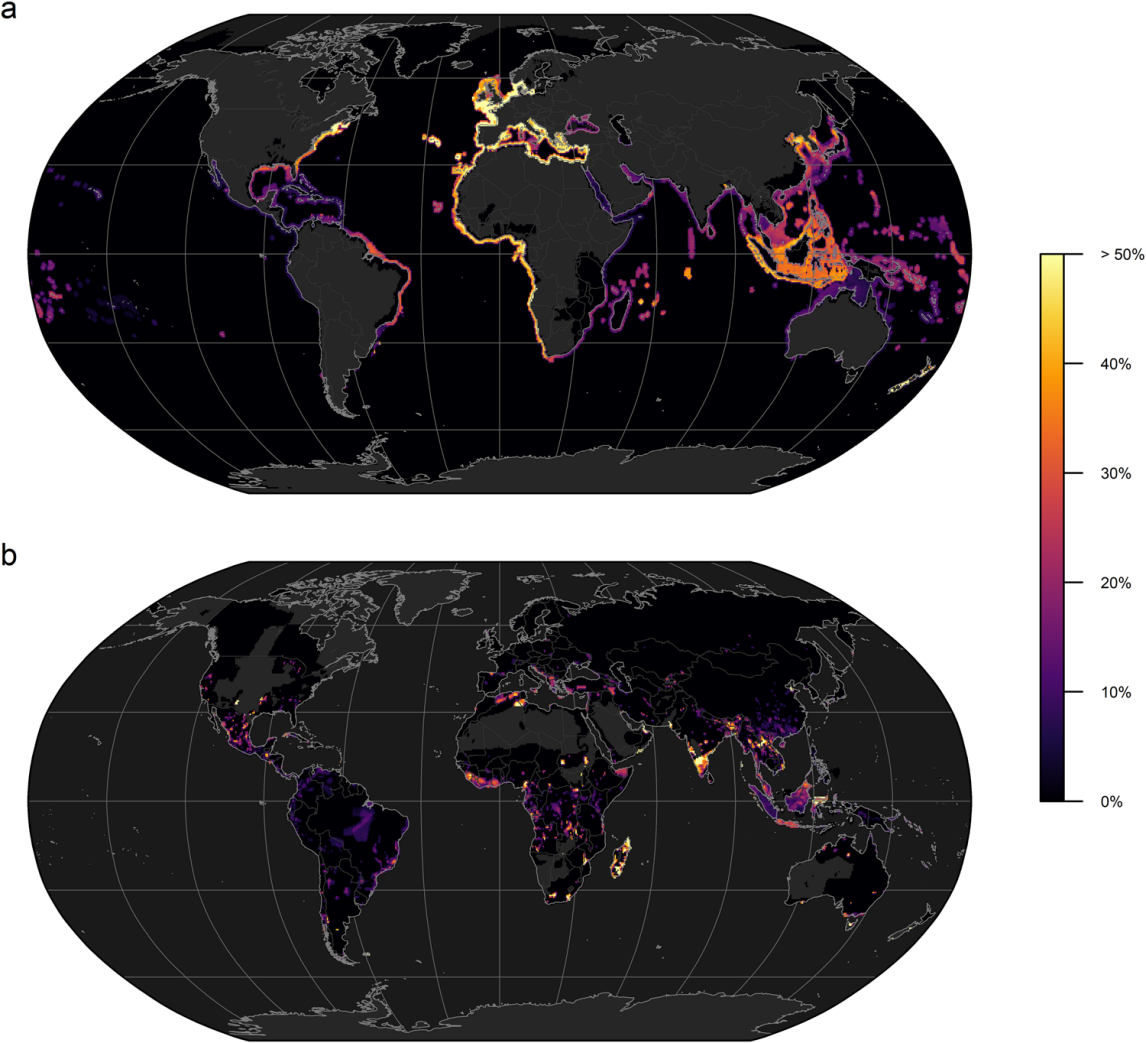
Potentially threatened fraction of data deficient species. Fraction of Data Deficient species (*n* = 7699, IUCN Version 2020-3) predicted to be threatened by extinction for marine (**a**) and non-marine species (**b**) according to our machine learning classifier.

On land, these likely threatened DD species are scattered across all continents and are often geographically restricted to smaller ranges (Fig. 2b; Supplementary Fig. 3), such as in central Africa, Madagascar and southern Asia. The greatest number of threatened marine DD species are found in south-eastern Asia, followed by the eastern Atlantic coastline as well as numerous atolls and islands (Supplementary Fig. 4). In fact, between a third and half of marine DD species around the world’s coastlines were predicted to be threatened by extinction, most notably along the eastern Atlantic coastline including the Mediterranean basin (Fig. 2a; Supplementary Fig. 3).

In addition to roughly 40% of Data Deficient ray-finned fishes (*Actinopterygii*), malacostracans (*Malacostraca*), bivalves, snails and slugs (*Gastropoda*), we found a staggering 960 out of 1130 (85%) Data Deficient amphibians (*Amphibia*), and more than half of Data Deficient anthozoans (*Anthozoa*; marine invertebrates including anemones and corals), insects (*Insecta*), mammals (*Mammalia*) and reptiles (*Reptilia*) likely to be threatened by extinction (Supplementary Table 1).

This is highly relevant for conservation and sustainability analyses, as some of these groups are amongst the most frequently considered ones^7^. More specifically, the classification of DD amphibians, mammals, and reptiles is likely to further increase both the absolute and relative number of species threatened by extinction in these taxonomic groups. For instance, an additional 14% of amphibians were predicted to be threatened by our ML classifier. This would raise the relative number of amphibian species being threatened by extinction from 39% to 47%. Similarly, the fraction of threatened mammals and reptiles likely increases when accounting for DD species (from 26% to 31% and 19% to 25%, respectively; Supplementary Table 1).

For selected species groups, models that suggest Red List categories or probabilities of being threatened for DD species exist, e.g., for amphibians^24^, reptiles^38^, terrestrial mammals^39^ or sharks and rays^43^. Howard and Bickford found 63% of DD amphibians to be threatened, mostly in South America, central Africa and North Asia, but also state that this is an underestimation^24^. Our model predicts 85% of DD amphibians to be threatened. Bland and Böhm identified 19% out of 292 DD terrestrial reptile species as threatened^38^, while our model identified 59% of reptiles as threatened, but we include over 1000 species and terrestrial, freshwater and marine species, the latter of which are thought to be more likely to be threatened^47^. The regions for conservation priorities for both reptiles and amphibians match those previously found, which are congruent with known hotspots for threatened species^38^. A previous assessment for terrestrial mammals identified 64% of DD terrestrial mammals as threatened^39^, while our model classifies 61% of DD terrestrial and marine mammals as threatened. Sharks and rays in the Mediterranean and North East Atlantic were modelled to contain 62% and 55% threatened species, respectively^44^. On a global scale, we found 26% of DD species in this group to be threatened (Supplementary Table 1). This is concordant with Dulvy et al., which found every fourth species of the ray and shark family to be threatened with extinction and who found the Mediterranean to be a hotspot for extinction^48^, explaining the large discrepancy of the local values to our global one.

### Data-deficiency causes regionally biased conservation priorities

The high variance found in the predicted probabilities of being threatened by extinction (i.e., PE scores) at the species level implies that more accurate assessments of DD species could shift regional conservation priorities. We predicted higher PE scores for DD than for DS species in most regions of the world (Supplementary Fig. 5), suggesting that current conservation concerns could, in fact, be underestimated. In marine systems, however, this seems to be restricted to coastal waters as well as high latitudes.

**Fig. 3:**
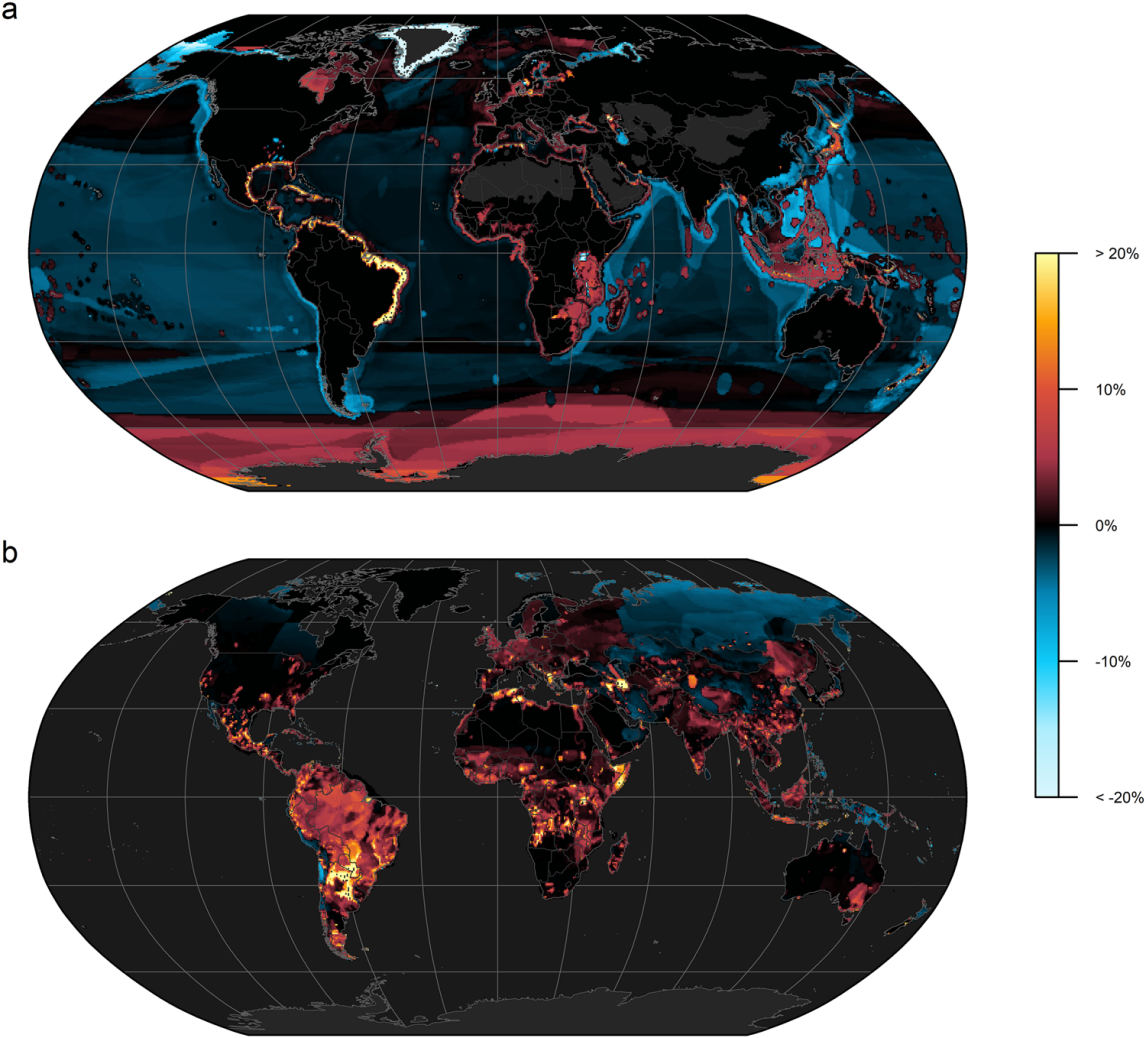
Data Deficient species change conservation priorities. Percent change in average PE score (i.e., predicted probability of being threatened by extinction) for marine (**a**) and non-marine species (**b**) following the inclusion of Data Deficient species alongside data-sufficient species.

DD species in marine systems seem to be most relevant around the world’s coastlines, as well as around temperate to tropical islands and atolls, but less relevant in international waters (Fig. 3a). For instance, we found an increase in average PE score by more than 20% once DD were considered alongside DS species in e.g., the Gulf of Mexico, the Caribbean and south America’s Atlantic coast (Fig. 3a). Even in biodiversity-rich regions the average PE score increased another 10% to 15% due to the extant DD species, such as in the Gulf of Guinea and South-eastern Asian seas. Here, numerous DD reef forming corals, sharks, rays, chimaeras, and marine fish species seem to be particularly relevant for a timely and expert-based threat assessment (Supplementary Figs. 3,  6). In contrast, including DD species did not change or even lowered the average PE score in large parts of international seas (Fig. 3a). Although marine biodiversity as we know it today is richest in coastal waters^49^, these results should be interpreted with caution because the underlying range maps for many marine species can be too coarse^50^, which may be especially true for DD species in international seas.

Furthermore, DD species on land (i.e., strictly non-marine species) seem to have the potential to regionally boost the conservation relevance in most of the world’s megadiverse countries^51^. Across Central to South America, we found a widespread increase of 10% to 20% in average PE score when including DD in addition to DS species (Fig. 3b). Notably, often only few taxonomic groups accounted for most of the observed increase in average PE score (Supplementary Fig. 6). For instance, the addition of predicted scores for DD amphibians, reptiles, mammals, rays and other freshwater groups in large parts of South America resulted in a widespread increase in average PE score, including for example the Amazon basin, the tropical Andes, the Atlantic Forest and Cerrado. However, these estimates are based on limited taxonomic groups and may be different if spatially explicit range maps for more taxa were available (e.g., plants).

In Africa, DD amphibians, reptiles, mammals, and freshwater ray-finned fishes (*Actinopterygii*) increased the average PE score locally across freshwater systems (e.g., Lake Victoria), tropical rainforests and savannas throughout the continent (Fig. 3b; Supplementary Fig. 6). We further discovered an increase in average PE score in numerous smaller isolated patches distributed around the world once DD extant species’ scores were acknowledged, such as in the Northern Territory and the Murray–Darling basin of Australia. Overall, the potential effects on PE score due to DD species were much more restricted to a regional level on land compared to marine systems, presumably due to spatially more explicit, and restricted, range maps for DD species on land.

## Conclusion

Previously, the risk of misjudging the importance of individual DD species outweighed the benefits of including them in Red List applications, resulting in regionally biased conservation prioritization. This study suggests that automatized classifiers built on species’ range maps and species observations can provide accurate and rapid pre-assessments on a large, global, and multitaxon scale. In contrast to previous approaches, our classifier is able to provide standardized predictions across multiple taxonomic groups^16^, making results between taxa directly comparable. The presented results show that DD species vary greatly in probability of being threatened by extinction, indicating a highly heterogenous bias that propagates into consequential Red List applications. As such, inferences built upon Red List-derived numbers of threatened species^30^ as well as numerically converted threat-levels^32^ could be biased. The generated predictions (i.e., PE scores) could facilitate the inclusion of DD species in sustainability-relevant applications^27^ and modelling approaches^26^. We encourage the extended use of our algorithm for screening for updates^14^ in the status of DS species, as well as large-scale pre-assessments of species not yet evaluated by the IUCN^42^ for a targeted completion of the IUCN Red List of Threatened Species.

## Methods

### Species data

We retrieved all spatial range map datasets (i.e., mammals, amphibians, reptiles, fish, marine groups, selected vascular plant groups and freshwater groups) available at the IUCN Red List (https://www.iucnredlist.org/resources/spatial-data-download, Version 2020-3)^45,46^ in March 2021, covering 44,924 species in the following taxonomic classes: *Actinopterygii*, *Agaricomycetes*, *Amphibia*, *Anthozoa*, *Aves*, *Bivalvia*, *Branchiopoda*, *Bryopsida*, *Cephalaspidomorphi*, *Charophyaceae*, *Chondrichthyes*, *Clitellata*, *Gastropoda*, *Hydrozoa*, *Insecta*, *Jungermanniopsida*, *Lecanoromycetes*, *Liliopsida*, *Lycopodiopsida*, *Magnoliopsida*, *Malacostraca*, *Mammalia*, *Myxini*, *Polypodiopsida*, *Reptilia* and *Sarcopterygii*. Range maps for bird species were not downloaded separately, because of their limited number of DD species. Species taxonomy, native countries, environmental domain (i.e., the occurrence in terrestrial, freshwater, marine systems and combinations thereof) and Red List category were available from IUCN for all species, i.e., Least Concern (LC), Lower Risk/Least Concern (LR/LC), Lower Risk/Conservation Dependent (LR/CD), Near Threatened (NT), Vulnerable (VU), Endangered (EN), Critically Endangered (CR), Extinct (EX), Extinct in the Wild (EW) and Data Deficient (DD). The spatial dataset consists of seasonal range maps (i.e., for each species one or several range maps out of “resident”, “breeding season”, “non-breading season”, “passage”, and “seasonal occurrence uncertain” were available). Only those range maps labelled as “native” and “extant” and only species that were not categorized as EW or EX were considered (*n* = 44,908 species).

### Predictor data

The correlate variables are summarized in Supplementary Table 2. Species taxonomy (i.e., taxonomic kingdom, phylum, and class) was included as potential predictor and surrogate for phylogenetic data^42^. Habitat preferences were retrieved from the Red List using *rredlist*^52^ in R. Occupied types of habitats as well as the number of different types of habitats, sub-habitats, and habitats of major importance were included as predictor. Occurrence data was retrieved from the Global Biodiversity Information Facility (GBIF)^53^ and the Ocean Biodiversity Information System (OBIS)^54^ using their corresponding application programming interfaces via the packages *rgbif*^55^ and *robis*^56^ in R. We only considered occurrence data that were collected between the years 2010 and 2020. For each species, we retrieved the maximum number of occurrence points per native country from GBIF (i.e., 100,000 data points per request), and for marine species, we additionally downloaded all data available from OBIS. The total number of occurrence points as well as the number of occurrence cells in a global grid (0.5-degree cells) was counted.

Because environmental threats can vary considerably across space and we expect the species to be exposed heterogeneously within their ranges, we extracted mean, minimum, maximum, and median values of environmental stressors and features across each species’ seasonal range map as well as its occurrence cells.

The included features were representative for the major drivers of biodiversity change, i.e. climate change, habitat change, overexploitation, invasive species and pollution^57^. As climatic dataset we retrieved all CHELSA bioclimatic variables^58,59^. The European Space Agency’s land cover product for the year 2015 in 300 m resolution^60^ was used to calculate fractions for different natural land cover types (*n* = 17). One raster was calculated per land cover class, representing the proportion of land covered by that class per cell. As general indicators of anthropogenic land use and land use change we included the global human footprint index^61^, including associated stressors such as population density, cropland area and pasture area, human modification index^62^, future urban expansion probabilities^63^, fraction of land designated to protected areas^64^, deforestation rates between the years 2000 and 2019^65^, different habitat heterogeneity metrics^66^ and cumulative application rates of different pesticides^67^. We counted the number of power plants^68^ and dams^69^ within each species geographical range, and included country-specific water scarcity estimates^70^, annual streamflow^71^, stream connectivity indices^72^ as well as freshwater environmental variables^73^, including eutrophication, pollution and upstream land use fractions, to account for the most severe impacts in freshwater systems^74,75^. Illegal hunting activities remain problematic for many species^76^. Yet, to the best of our knowledge, global poaching data does not exist. Therefore, we included factors that may affect the rate of poaching on a global scale^77,78^, i.e., the human development index (HDI) in 2019, the average annual HDI growth between 1990–2019^79^ and the corruption perceptions index (CPI) in 2020 at country-level^80^. We further included estimated threats from species invasions, country-specific capacities to respond to invasion^81^, a set of modelled impacts on marine ecosystems^82,83^ and marine environmental variables^84,85^. All layers were aggregated for computational efficiency by averaging to 0.5-degree cells (approximately 56 km at the equator).

### Machine learning classifier

We aimed to estimate the probability of being threatened by extinction (hereafter: PE score) for DD species by training a machine learning classifier, fitted using species with known threat-levels. All DS species were reclassified into two groups based on their IUCN Red List categories: threatened by extinction (i.e., all species in the categories VU, EN, and CR) and not threatened by extinction (i.e., all species in the categories LC, LR/LC, LR/CD and NT). Species classified as DD (*n* = 7699) were set aside and not used for training or testing the classifier. All assessments identified by the IUCN as in need of an update were removed^16^, with one exception: if fewer than five records remained for a given taxonomic class, outdated assessment were kept to maximize the amount of training data. We used a data split for model validation^16,39,86,87^. Therefore, the remaining dataset (*n* = 28,363 species) was split into training (75%) and testing (25%) data. During the data split the balance of threat categories were maintained within both taxonomic families and environmental domains, i.e., marine and non-marine.

We used different partitions of the dataset to train ML classifiers in two ways: (1) all species together, and (2) separate classifiers for marine and non-marine species to account for the different spatial extents of the predictor data. For each data partition, we utilized a set of machine learning methods suitable for classification problems, each with its own strengths and weaknesses^88^. The best performing data partition (i.e., partition 1; for all species) was selected based on the highest average AUC (see section Model evaluation) across all taxonomic groups. Although irrelevant covariates tend to be automatically ignored in the utilized algorithms^89–92^, a smaller set of covariates can improve performance and increase interpretability of the model. Therefore, we performed feature selection on the training data of each partition by using the Boruta algorithm^93^. This algorithm compares the original feature importance to the importance of random shadow features while accounting for possible correlations and interactions. All features considered relevant at the 99% confidence level after 50 runs of the algorithm were kept (i.e., 270 features in partition 1). NA-values were imputed with random values using the package *Hmisc*^94^ in R, i.e., about 5% of the values in the remaining features. Optimal model settings and parameters were selected using the AutoML function in H2O.ai^89,90^. We used 10-fold cross validation for calibrating all models (e.g., tuning hyperparameters). In addition, the two classes (i.e., threatened versus not threatened species) were balanced during cross validation by oversampling of the smaller class (i.e., threatened species). In partition 1, a total of 220 models (i.e., base-learners) was trained, including generalized linear models, random forests, gradient boosted classification trees, deep neural networks and an extremely randomized forest (details in reference^90^). Ultimately, a so-called super-learner^95^ was generated using a non-negative generalized linear model with regularization (least absolute shrinkage and selection operator) to produce more sparse ensembles^90^, combining the best features of the trained base-learners into one superior model. In total, 23 base-learners contributed to the predictions of the super-learner (Supplementary Table 3).

### Model evaluation

The performance of all base-learners and the super-learner of the best performing data partition (i.e., partition 1; trained using all species) was assessed using the set aside testing data (*n* = 6857 species). In addition, we assessed model performance using DD species that have been re-evaluated and assigned a threat category in Red List Version 2021-2 (*n* = 123 species)^15^.

We calculated accuracy as the fraction of correctly classified species across the total number of species (Eq. 1), specificity as the fraction of not threatened species being correctly classified as not threatened (Eq. 2), sensitivity (i.e., recall) as the fraction of threatened species being correctly classified as threatened (Eq. 3), the false positive rate as fraction of not threatened species being classified as threatened (Eq. 4), the negative predictive value as the fraction of not threatened species across species predicted to be not threatened (Eq. 5), the positive predictive value (i.e., precision) as the fraction of threatened species across species predicted to be threatened (Eq. 6) and, balanced accuracy as the average of specificity and sensitivity.

1 $${Accuracy}=\frac{{True}\,{Positive}+{True}\,{Negative}}{{True}\,{Positive}+{False}\,{Negative}+{True}\,{Negative}+{False}\,{Positive}}$$

2 $${Specificity}=\frac{{True}\,{Negative}}{{True}\,{Negative}+{Fals}e\,{Positive}}$$

3 $${Sensitivity}=\frac{{True}\,{Positive}}{{True}\,{Positive}+{False}\,{Negative}}$$

4 $${False}\,{positive}\,{rate}=\frac{{False}\,{Positive}}{{False}\,{Positive}+{True}\,{Negative}}$$

5 $${Negative}\,{predictive}\,{value}=\frac{{True}\,{Negative}}{{True}\,{Negative}+{False}\,{Negative}}$$

6 $${Positive}\,{predictive}\,{value}=\frac{{True}\,{Positive}}{{True}\,{Positive}+{False}\,{Positive}}$$

In addition, AUC, AUC_PR_ and GINI coefficient were calculated^89,90^ as threshold-independent performance measures for binary classifiers. A value of 1 depicts the highest performance for all metrics. AUC is the area under the receiver operating characteristic curve for sensitivity (Eq. 3) versus false positive rate (Eq. 4). This measure is influenced by correctly assigned species as being not threatened (True Negatives), which is the dominating class in our dataset. In contrast, AUC_PR_, as the area under the receiver operating characteristic curve for precision (Eq. 6) versus recall (Eq. 3), is not affected by true negatives (i.e., correctly predicted not-threatened species) but instead affected by how precise the classifier is in predicting which species are threatened. The GINI coefficient describes the degree of separation between both classes (i.e., threatened versus not threatened), with a value of 1 indicating perfect separation.

Permutation variable importance was calculated as the performance loss (i.e., in AUC) on the testing data before and after a feature was permuted. Features were permuted one at a time in a total of 50 repetitions. In partition 1, the species’ taxonomic affiliation, proxies for geographic range size (i.e., number of native countries, species range extent and number of occurrence cells), anthropogenic activities within the species’ range (number of dams, road density, number of powerplants, human footprint index), and occupied environmental domains (combinations of terrestrial, freshwater and marine) are most important for the super-learner in accurately separating not threatened and threatened species (Supplementary Fig. 7).

### Statistics and reproducibility

Analyses were conducted using R version 4.0.3^96^ in RStudio version 1.4.1103^97^. Data were obtained from GBIF, OBIS and IUCN using the packages *rgbif, robis, and rredlist*^52,55,56^. Handling of spatial and other data was conducted using the R packages *caTools*, *doParallel*, *exactextractr*, *fasterize*, *maptools*, *parallel*, *raster*, *readxl*, *rgdal*, *rgeos*, *sf*, *sp*, *stringr*, *tidyverse*, and *xlsx*^96,98–110^, and in python using the arcpy module from ArcGIS Pro version 2.9.0^111^. Machine learning algorithms were trained and evaluated using the H2O.ai interface (Version 3.36.0.4) for R^89^ and *caret*^112^. Figures were created using *ggplot*^113^, *ggridges*^114^, *rnaturalearth*^115^, *viridis*^116^ and base R^96^.

### Reporting summary

Further information on research design is available in the Nature Research Reporting Summary linked to this article.

## Supplementary information

Supplementary Information

Description of Additional Supplementary Files

Supplementary Data 1

Supplementary Data 2

Supplementary Data 3

Reporting Summary

## Data Availability

Previously published and open-access source data were retrieved from refs. ^45,46,53,54,58–73,79–84^. All data generated in this study is available without restrictions. The generated predictions for the testing data, complete dataset and updated Data Deficient species are provided as supplementary files (Supplementary Data 1–3). Any further requests can be directed to the corresponding author.

## Code availability

All code generated in this study is available without restrictions. R code for preparing the data, for training and testing the ML classifier, as well as applying the algorithm is available on GitHub (https://github.com/jannebor/dd_forecast)^117^. Although functionality may be given in other version, the code in this study was used in R version 4.0.3^96^ in RStudio version 1.4.1103^97^. The classifier can be applied for single species using our web application (https://ml-extinctionrisk.indecol.no/). Any further requests can be directed to the corresponding author.
