# Supplementary material for: More than half of data deficient species predicted to be threatened by extinction
Source: Commun Biol. 2022 Aug 4;5:679. doi: 10.1038/s42003-022-03638-9 (PMC9352662; doi:10.1038/s42003-022-03638-9)
Supplement: Supplementary file 1 — Supplementary Information [file 42003_2022_3638_MOESM1_ESM.pdf]

More than half of Data Deficient species predicted to be threatened by extinction

Jan Borgelt[1], Martin Dorber[1], Marthe Alnes Høiberg[1], Francesca Verones[1]

1. Industrial Ecology Programme, Department of Energy and Process Engineering, Norwegian University of Science and Technology (NTNU), Trondheim, Norway

corresponding author: Jan Borgelt (jan.borgelt@ntnu.no)

Supplementary Information

This file includes Supplementary Figures 1-7 and Supplementary Tables 1-3

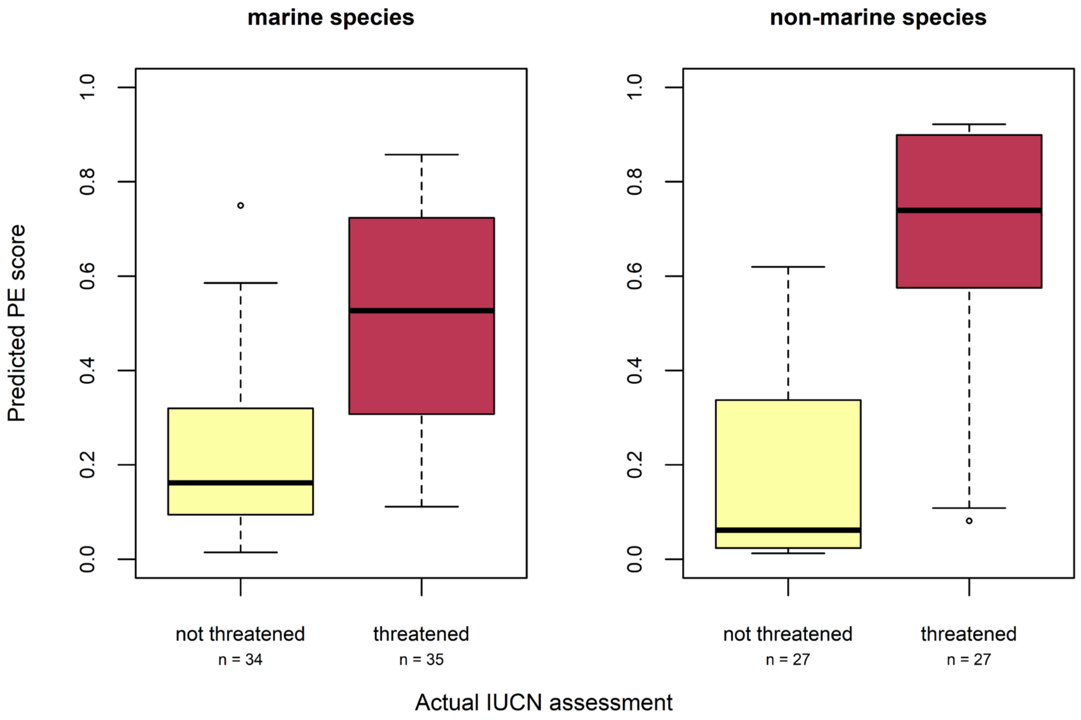

Supplementary Figure 1: Boxplot showing the interquartile range (box), median (black line), minimum and maximum values without outliers (error bars), and outliers (points) of predicted probability of being threatened by extinction (i.e., PE score) of formerly Data Deficient species (n = 123 Data Deficient in IUCN Version 2020-3)[1,2] *across updated IUCN assessments (updated in Version 2021-2 to either not threatened or threatened)*[3].

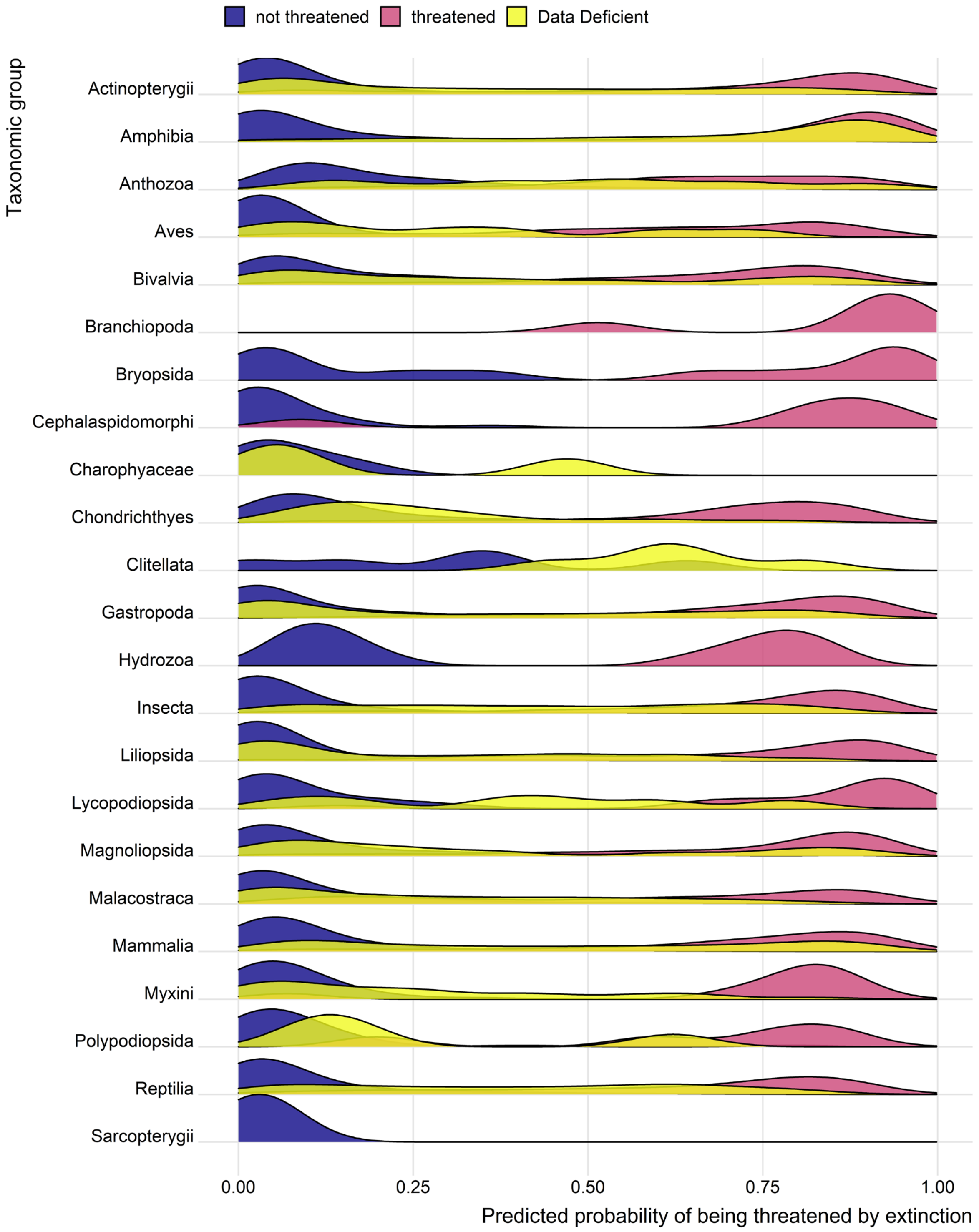

Supplementary Figure 2: Frequency distribution of predicted PE scores for not threatened (blue), threatened (red) and Data Deficient (yellow) species per taxonomic class of the spatial dataset of the IUCN Red List of threatened species (Version 2020-3)[1,2].

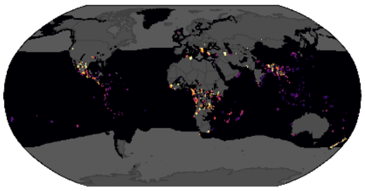
Actinopterygii

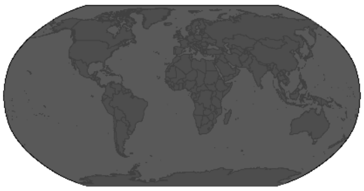
Agaricomycetes

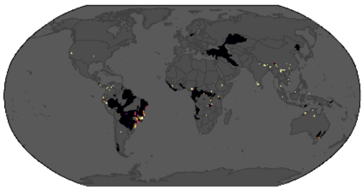
Amphibia

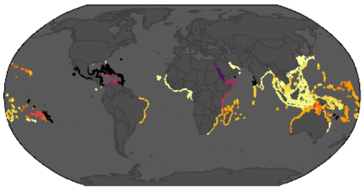
Anthozoa

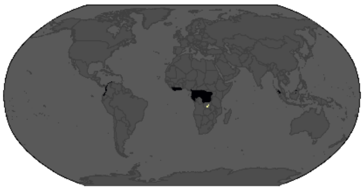
Aves

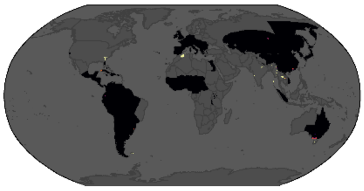
Bivalvia

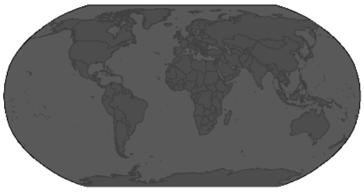
Branchiopoda

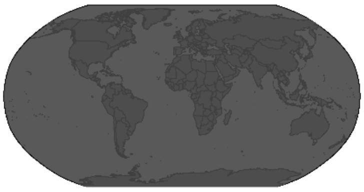
Bryopsida

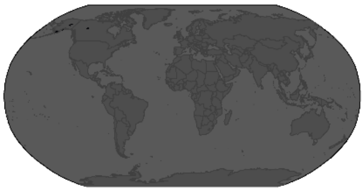
Cephalaspidomorphi

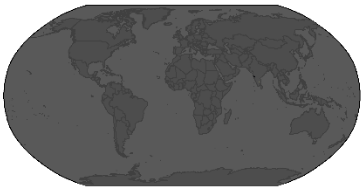
Charophyaceae

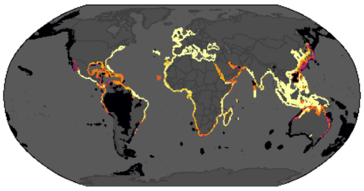
Chondrichthyes

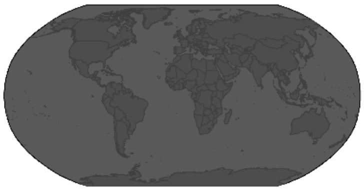
Clitellata

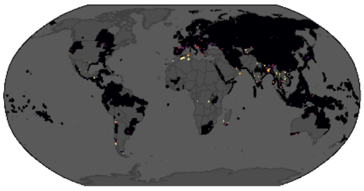
Gastropoda

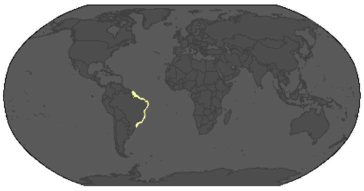
Hydrozoa

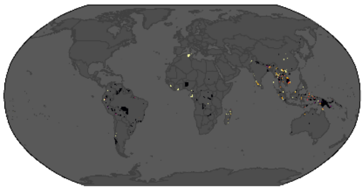
Insecta

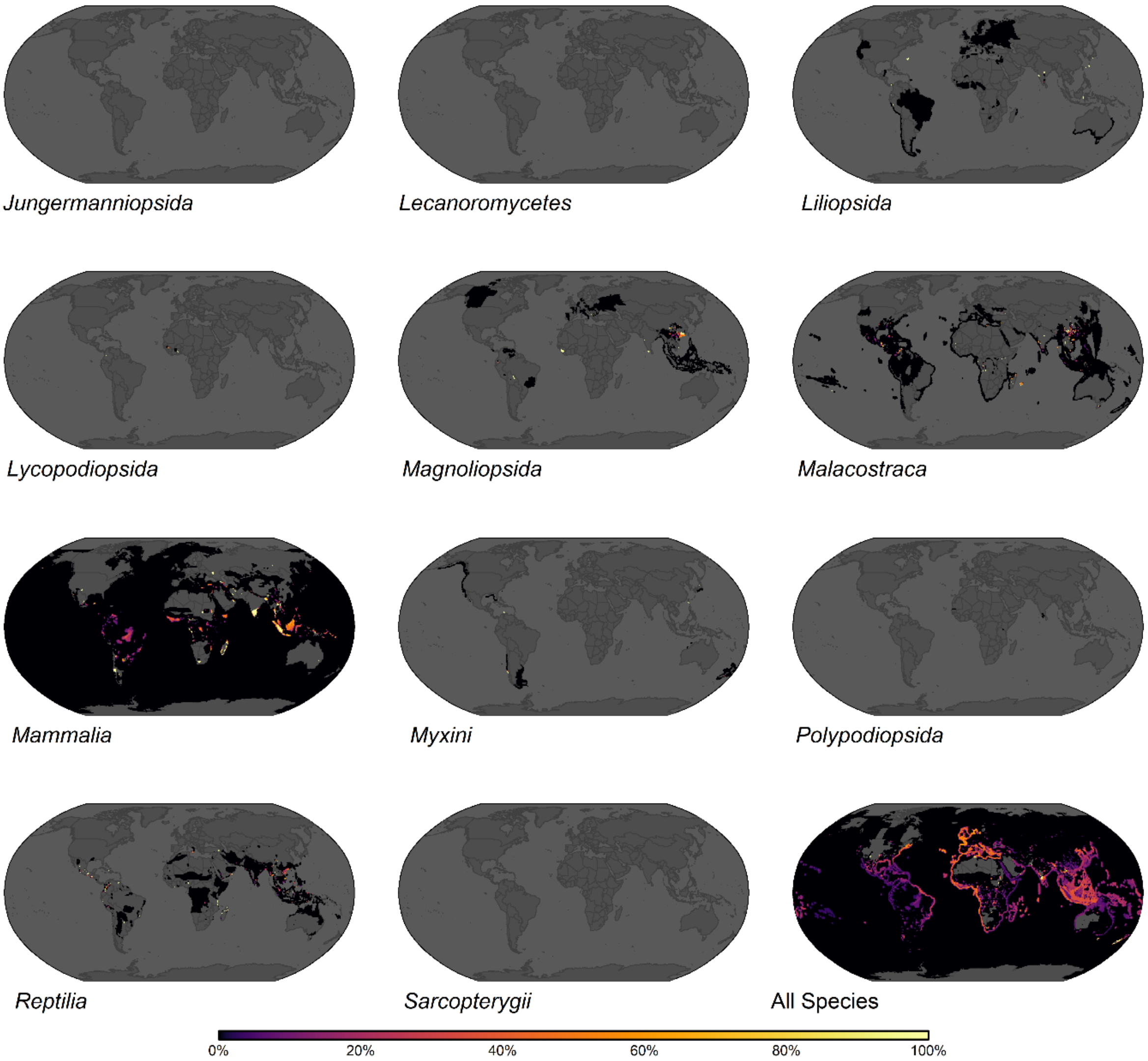

Supplementary Figure 3: Fraction of Data Deficient species predicted to be threatened by extinction per taxonomic class and for all Data Deficient species (n = 7,699) of the spatial dataset of the IUCN Red List of threatened species (Version 2020-3)[1,2].

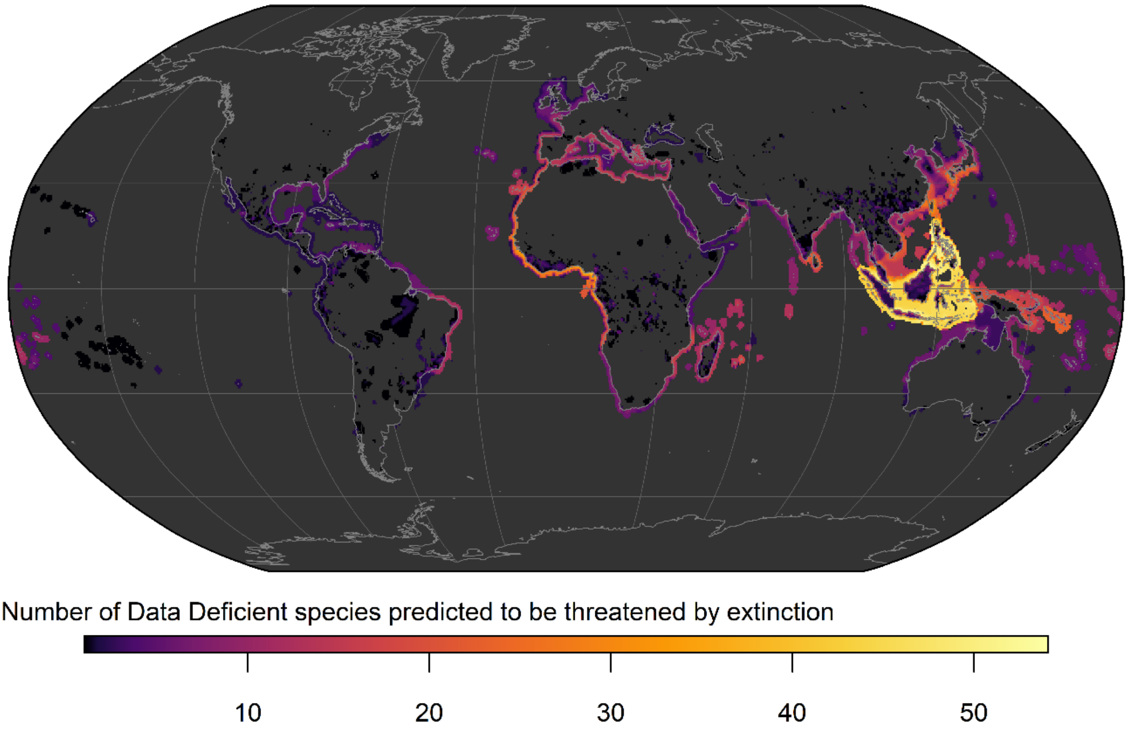

Supplementary Figure 4: Number of Data Deficient species of the spatial dataset of the IUCN Red List of threatened species (Version 2020-3)[1,2] predicted to be threatened by extinction across the world.

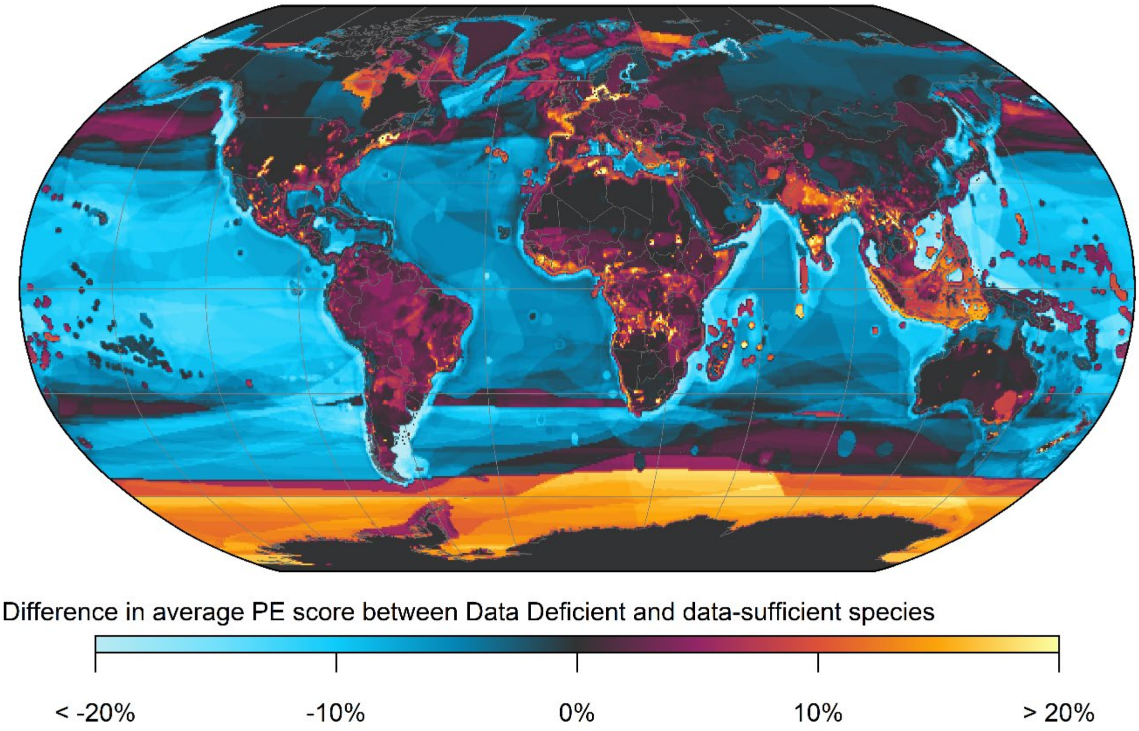

Supplementary Figure 5: Regional differences in average PE score between Data Deficient and data-sufficient species of the spatial dataset of the IUCN Red List of threatened species (Version 2020-3)[1,2].

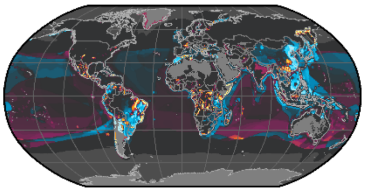

Actinopterygii

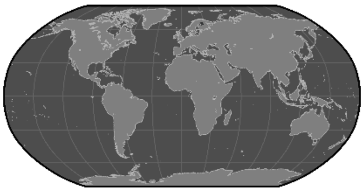

Agaricomycetes

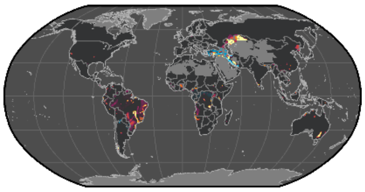

Amphibia

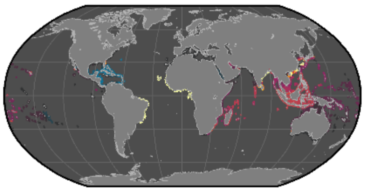

Anthozoa

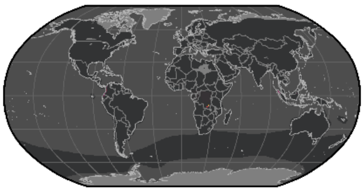

Aves

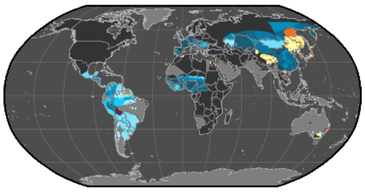

Bivalvia

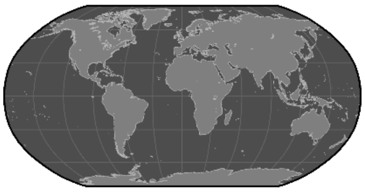

Branchiopoda

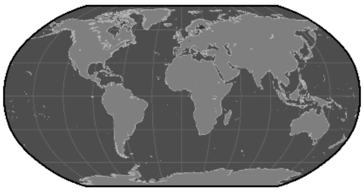

Bryopsida

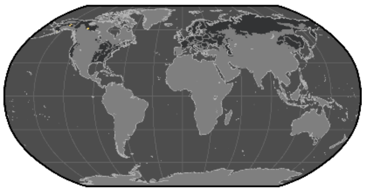

Cephalaspidomorphi

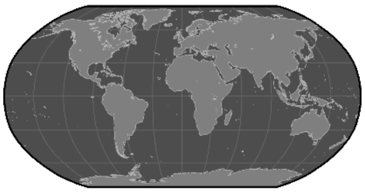

Charophyaceae

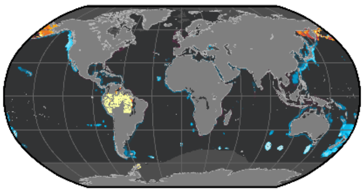

Chondrichthyes

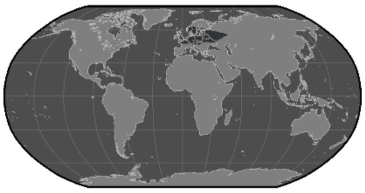

Clitellata

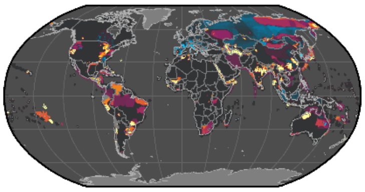

Gastropoda

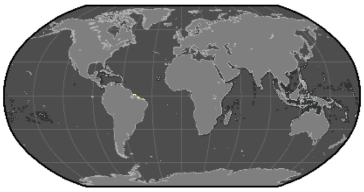

Hydrozoa

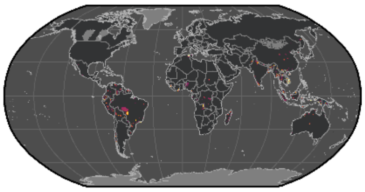

Insecta

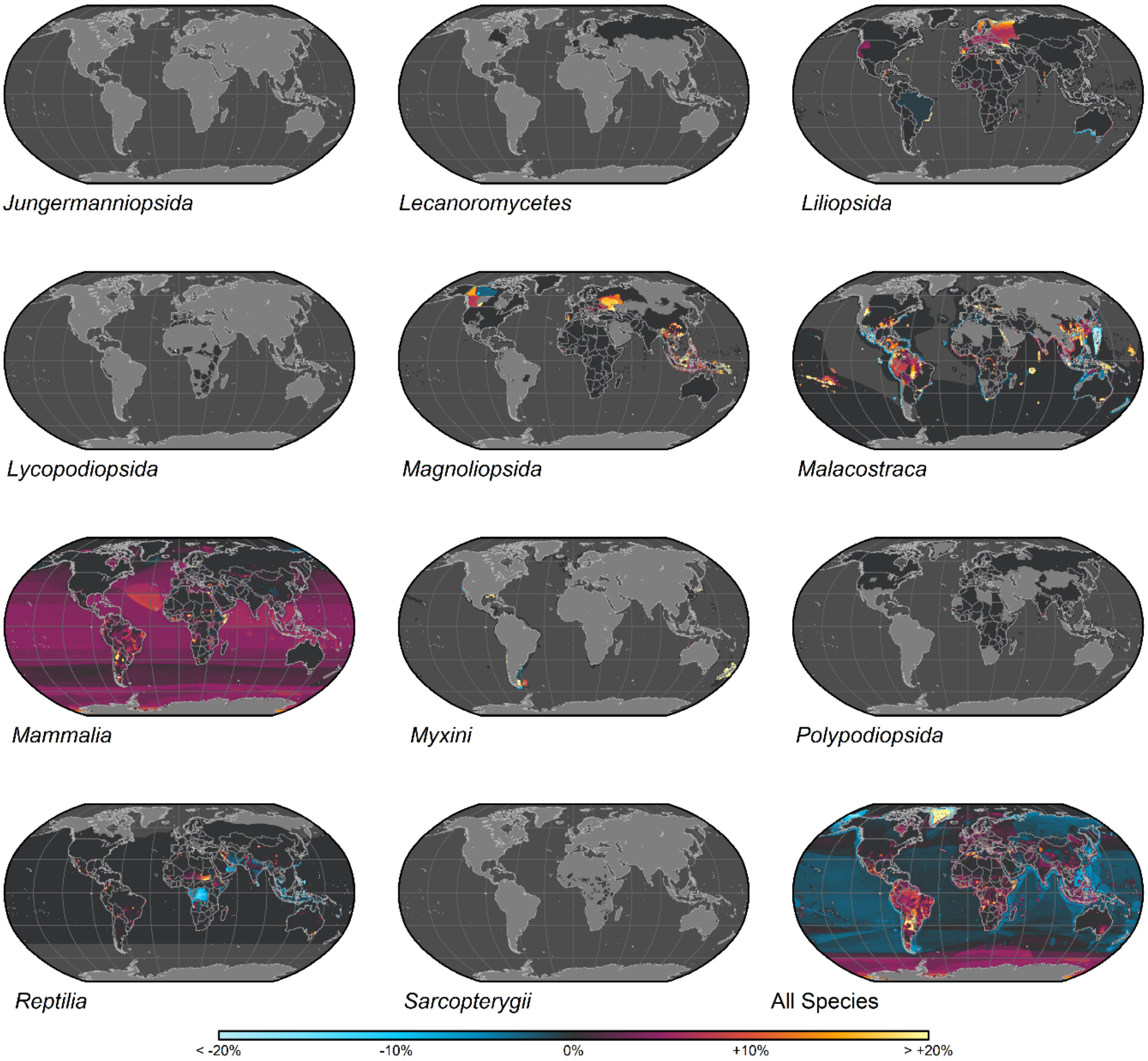

Supplementary Figure 6: Percent change in average PE score when Data Deficient species are considered along data-sufficient species for all taxonomic groups and all species (n = 44,908) of the spatial dataset of the IUCN Red List of threatened species (Version 2020-3)[1,2].

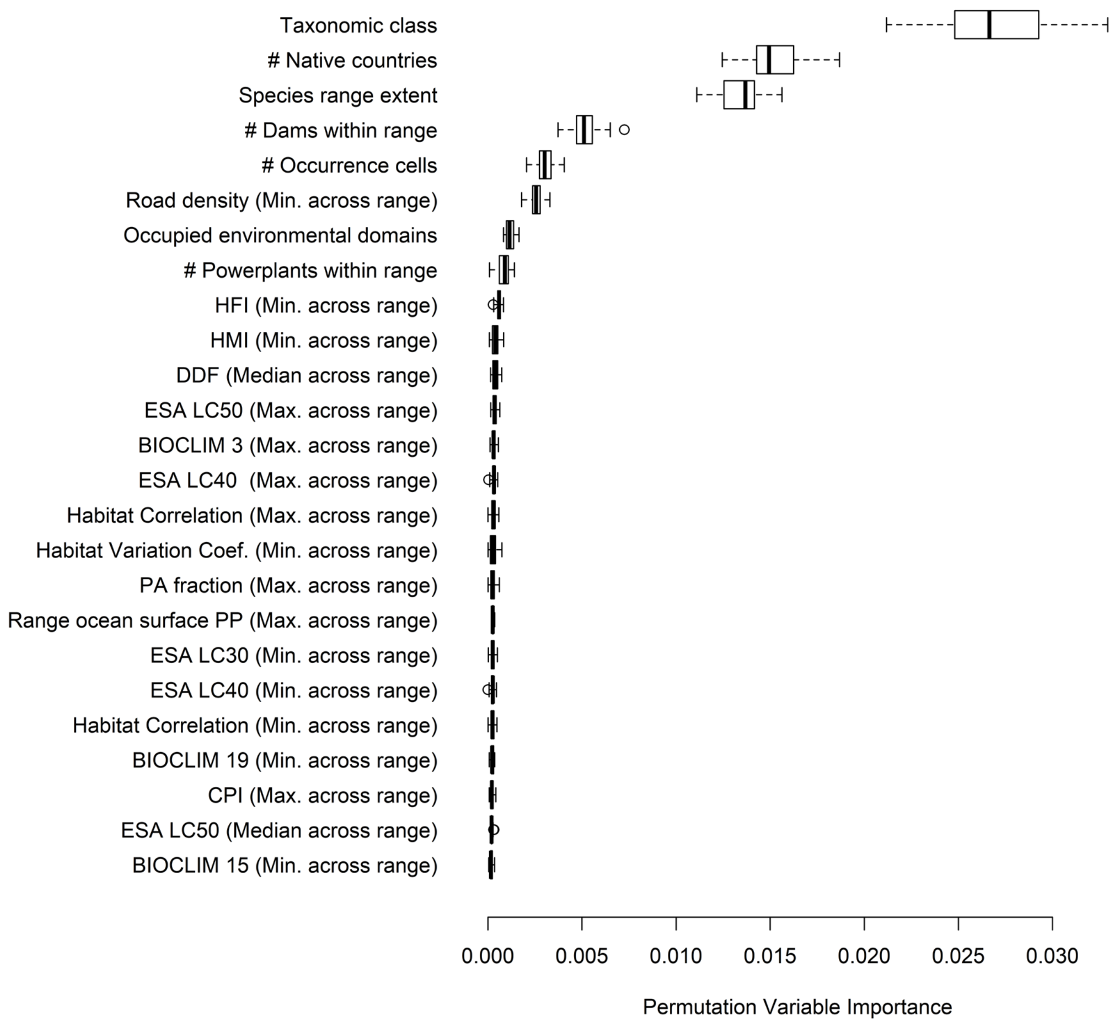

Supplementary Figure 7: Boxplot showing the interquartile range (box), median (black line), minimum and maximum values without outliers (error bars), and outliers (points) of permutation variable importance for the top 25 variables of the presented classifier, based on performance loss (AUC) during 50 runs of feature permutation. Abbreviations: Human Footprint Index (HFI), Human Modification Index (HMI), demersal destructive fishing (DDF), ESA LC (European Space Agency Land Cover, 30 = Mosaic cropland (>50%) / natural vegetation (tree, shrub, herbaceous cover) (<50%); 40 = Mosaic natural vegetation (tree, shrub, herbaceous cover) (>50%) / cropland (<50%); 50 = Tree cover, broadleaved, evergreen, closed to open (>15%)), BIOCLIM (3 = isothermality; 15 = precipitation seasonality; 19 = mean monthly precipitation amount of the coldest quarter), Protected Area (PA), Primary productivity (PP), Corruption Perception Index (CPI).

*Supplementary Table 1: Predictions across taxonomic groups. Total number of species and number of species (*predicted to be, PE cut-off: 0.388) threatened by extinction across data-sufficient and Data Deficient species for each taxonomic class. Classifier performance (i.e., AUC, as well as Accuracy (Acc.), Sensitivity (Sens.), and Specificity (Spec.)) based on the testing dataset (25%). Note: Performance metrics were calculated for taxonomic classes only if both categories (i.e., threatened vs. not threatened) present in testing data.*

	Data-sufficient		Data Deficient		Performance			
Taxonomic class	total	threatened[2]	total	threatened*	AUC	Acc.	Sens.	Spec.
Actinopterygii	9404	2134 (23%)	1876	880 (47%)	0.93	0.86	0.80	0.88
Amphibia	5801	2268 (39%)	1130	960 (85%)	0.93	0.85	0.93	0.79
Anthozoa	691	227 (33%)	135	98 (73%)	-	-	-	-
Aves	1803	179 (10%)	7	3 (43%)	0.85	0.92	0.40	0.97
Bivalvia	329	72 (22%)	77	37 (48%)	0.83	0.82	0.64	0.87
Branchiopoda	5	5 (100%)	0	0	-	-	-	-
Bryopsida	12	6 (50%)	1	1 (100%)	1.00	0.67	1.00	0.00
Cephalaspidomorphi	27	6 (22%)	1	0 (0%)	0.33	0.75	0.00	1.00
Charophyceae	8	0 (0%)	3	1 (33%)	-	-	-	-
Chondrichthyes	928	309 (33%)	222	58 (26%)	0.85	0.78	0.75	0.79
Clitellata	5	0 (0%)	5	5 (100%)	-	-	-	-
Gastropoda	1839	608 (33%)	551	260 (47%)	0.95	0.90	0.82	0.92
Hydrozoa	14	5 (36%)	2	2 (100%)	-	-	-	-
Insecta	2293	415 (18%)	882	546 (62%)	0.92	0.86	0.85	0.86
Lecanoromycetes	2	1 (50%)	0	0	-	-	-	-
Liliopsida	542	83 (15%)	31	13 (42%)	0.98	0.95	0.93	0.96
Lycopodiopsida	28	15 (54%)	6	4 (67%)	-	-	-	-
Magnoliopsida	542	180 (33%)	55	23 (42%)	0.86	0.79	0.74	0.82
Malacostraca	1380	310 (22%)	832	335 (40%)	0.89	0.75	0.89	0.69
Mammalia	4962	1293 (26%)	818	495 (61%)	0.86	0.83	0.70	0.87
Myxini	46	9 (20%)	29	9 (31%)	-	-	-	-
Polypodiopsida	41	5 (12%)	4	1 (25%)	-	-	-	-
Reptilia	6500	1263 (19%)	1032	605 (59%)	0.89	0.84	0.67	0.87
Sarcopterygii	5	1 (20%)	0	0	-	-	-	-

*Supplementary Table 2: Complete list of data used as correlates in the generated machine learning classifier. *If applicable, variables were generated by retrieving mean, median, minimum, and maximum values of the corresponding layers across species range maps and occurrence cells, or native countries.*

Data	Layers	Variables*
IUCN Red List of threatened species[2]	35	35
Global Biodiversity Information Facility (GBIF)[4] & Ocean Biodiversity Information System (OBIS)[5]	5	5
Climatologies at high resolution for the earth's land surface areas[6]	19	152
ESA Land Cover CCI[7]	17	136
Global terrestrial Human Footprint maps for 1993 and 2009[8]	6	48
Managing the middle: A shift in conservation priorities based on the global human modification gradient[9]	1	8
Global forecasts of urban expansion to 2030 and direct impacts on biodiversity and carbon pools[10]	1	8
Protected Planet: The World Database on Protected Areas (WDPA)[11]	1	8
High-Resolution Global Maps of 21st-Century Forest Cover Change[12]	1	8
A global, remote sensing-based characterization of terrestrial habitat heterogeneity for biodiversity and ecosystem modelling[13]	14	112
PEST-CHEMGRIDS, global gridded maps of the top 20 crop-specific pesticide application rates from 2015 to 2025[14]	1	8
A Global Database of Power Plants[15]	1	1
GOODD, a global dataset of more than 38,000 georeferenced dams[16]	1	1
The WULCA consensus characterization model for water scarcity footprints: assessing impacts of water consumption based on available water remaining (AWARE)[17]	2	8
FLO1K, global maps of mean, maximum and minimum annual streamflow at 1 km resolution from 1960 through 2015[18]	56	448
Impacts of current and future large dams on the geographic range connectivity of freshwater fish worldwide[19]	2	2
Near-global freshwater-specific environmental variables for biodiversity analyses in 1 km resolution[20]	87	696
The Next Frontier: Human Development and the Anthropocene[21]	2	8
Corruption Perceptions Index[22]	1	4
Global threats from invasive alien species in the twenty-first century and national response capacities[23]	8	56
A Global Map of Human Impact on Marine Ecosystems[24]	18	144
Bio-ORACLE v2.0: Extending marine data layers for bioclimatic modelling[25,26]	161	1288

Supplementary Table 3: Classifier contributions. Weights of all (non-zero) models contributing to the super-learner, i.e., gradient boosted classification trees (GBM) and neural networks (DeepLearning).

Base-learner	Relative Importance	Percentage
GBM_grid_1_AutoML_1_model_45	0.337	0.157
GBM_grid_1_AutoML_1_model_34	0.202	0.094
GBM_grid_1_AutoML_1_model_39	0.194	0.090
GBM_grid_1_AutoML_1_model_25	0.167	0.077
GBM_grid_1_AutoML_1_model_60	0.160	0.075
GBM_1_AutoML_1	0.154	0.071
GBM_grid_1_AutoML_1_model_48	0.140	0.065
GBM_grid_1_AutoML_1_model_31	0.126	0.058
GBM_grid_1_AutoML_1_model_71	0.125	0.058
GBM_grid_1_AutoML_1_model_27	0.100	0.046
GBM_grid_1_AutoML_1_model_5	0.072	0.034
GBM_grid_1_AutoML_1_model_33	0.071	0.033
GBM_grid_1_AutoML_1_model_51	0.063	0.029
GBM_grid_1_AutoML_1_model_10	0.056	0.026
GBM_grid_1_AutoML_1_model_1	0.050	0.023
GBM_grid_1_AutoML_1_model_2	0.038	0.018
DeepLearning_grid_1_AutoML_1_model_35	0.023	0.011
GBM_grid_1_AutoML_1_model_17	0.022	0.010
DeepLearning_grid_1_AutoML_1_model_9	0.017	0.008
GBM_grid_1_AutoML_1_model_4	0.013	0.006
GBM_grid_1_AutoML_1_model_62	0.012	0.006
DeepLearning_grid_1_AutoML_1_model_13	0.009	0.004
GBM_grid_1_AutoML_1_model_46	0.000	0.000

Supplementary References

1. IUCN. Species Information Service. Version 2020-3. https://www.iucnredlist.org/resources/spatial-data-download (2021).
2. IUCN. The IUCN Red List of Threatened Species. Version 2020-3. https://www.iucnredlist.org (2020).
3. IUCN. The IUCN Red List of Threatened Species. Version 2021-2. https://www.iucnredlist.org (2021).
4. Chamberlain, S. *et al. rgbif: Interface to the Global Biodiversity Information Facility API. R package version 3.6.0.* https://cran.r-project.org/package=rgbif (2021).
5. Provoost, P. & Bosch, S. robis: Ocean Biodiversity Information System (OBIS) Client. R package version 2.3.9. https://CRAN.R-project.org/package=robis. (2020).
6. Karger, D. N. *et al.* Data from: Climatologies at high resolution for the earth's land surface areas. *Dryad, Dataset* https://doi.org/10.5061/dryad.kd1d4 (2018).
7. ESA. Land Cover CCI Product User Guide Version 2. Tech. Rep. http://maps.elie.ucl.ac.be/CCI/viewer/download.php (2017).
8. Venter, O. *et al.* Global terrestrial Human Footprint maps for 1993 and 2009. *Sci. Data* **3**, 160067 (2016).
9. Kennedy, C. M., Oakleaf, J. R., Theobald, D. M., Baruch-Mordo, S. & Kiesecker, J. Managing the middle: A shift in conservation priorities based on the global human modification gradient. *Glob. Chang. Biol.* **25**, 811–826 (2019).
10. Seto, K. C., Guneralp, B. & Hutyra, L. R. Global forecasts of urban expansion to 2030 and direct impacts on biodiversity and carbon pools. *Proc. Natl. Acad. Sci.* **109**, 16083–16088 (2012).
11. UNEP-WCMC & IUCN. Protected Planet: The World Database on Protected Areas (WDPA). *Cambridge, UK: UNEP-WCMC and IUCN* www.protectedplanet.net (2021).
12. Hansen, M. C. *et al.* High-Resolution Global Maps of 21st-Century Forest Cover Change. *Science (80-.).* **342**, 850–853 (2013).
13. Tuanmu, M. N. & Jetz, W. A global, remote sensing-based characterization of terrestrial habitat heterogeneity for biodiversity and ecosystem modelling. *Glob. Ecol. Biogeogr.* **24**, 1329–1339 (2015).
14. Maggi, F., Tang, F. H. M., la Cecilia, D. & McBratney, A. PEST-CHEMGRIDS, global gridded maps of the top 20 crop-specific pesticide application rates from 2015 to 2025. *Sci. Data* **6**, 170 (2019).
15. Byers, L. *et al.* A Global Database of Power Plants. *World Resour. Inst.* 1–18 (2019).
16. Mulligan, M., van Soesbergen, A. & Sáenz, L. GOODD, a global dataset of more than 38,000 georeferenced dams. *Sci. Data* **7**, 31 (2020).

17. Boulay, A.-M. *et al.* The WULCA consensus characterization model for water scarcity footprints: assessing impacts of water consumption based on available water remaining (AWARE). *Int. J. Life Cycle Assess.* **23**, 368–378 (2018).

18. Barbarossa, V. *et al.* Erratum: FLO1K, global maps of mean, maximum and minimum annual streamflow at 1 km resolution from 1960 through 2015. *Sci. Data* **5**, 180078 (2018).

19. Barbarossa, V. *et al.* Impacts of current and future large dams on the geographic range connectivity of freshwater fish worldwide. *Proc. Natl. Acad. Sci.* **117**, 3648–3655 (2020).

20. Domisch, S., Amatulli, G. & Jetz, W. Near-global freshwater-specific environmental variables for biodiversity analyses in 1 km resolution. *Sci. Data* **2**, 150073 (2015).

21. UNDP. *Human Development Report 2020. The Next Frontier: Human Development and the Anthropocene. New York. http://hdr.undp.org/en/content/human-development-report-2020*. (2020).

22. Transparency International. *Corruption Perceptions Index 2020*. (2020).

23. Early, R. *et al.* Global threats from invasive alien species in the twenty-first century and national response capacities. *Nat. Commun.* **7**, 12485 (2016).

24. Halpern, B. S. *et al.* A Global Map of Human Impact on Marine Ecosystems. *Science (80-.).* **319**, 948–952 (2008).

25. Assis, J. *et al.* Bio-ORACLE v2.0: Extending marine data layers for bioclimatic modelling. *Glob. Ecol. Biogeogr.* **27**, 277–284 (2018).

26. Tyberghein, L. *et al.* Bio-ORACLE: a global environmental dataset for marine species distribution modelling. *Glob. Ecol. Biogeogr.* **21**, 272–281 (2012).
