# Supplementary material for: More than half of data deficient species predicted to be threatened by extinction
Source: Commun Biol. 2022 Aug 4;5:679. doi: 10.1038/s42003-022-03638-9 (PMC9352662; doi:10.1038/s42003-022-03638-9)
Supplement: Supplementary file 2 — Description of Additional Supplementary Files [file 42003_2022_3638_MOESM2_ESM.pdf]

## Description of Additional Supplementary Files

**File Name:** Supplementary Data 1

**Description:** Predicted scores in the set-aside testing dataset, source data for Figure 1.

**File Name:** Supplementary Data 2

**Description:** Predicted scores for all species of the IUCN Red List Version 2020-3, source data behind the figures in the paper.

**File Name:** Supplementary Data 3
**Description:** Predicted scores for reclassified Data Deficient species, source data for Supplementary Figure 1.
