# Supplementary material for: More than half of data deficient species predicted to be threatened by extinction
Source: Commun Biol. 2022 Aug 4;5:679. doi: 10.1038/s42003-022-03638-9 (PMC9352662; doi:10.1038/s42003-022-03638-9)
Supplement: Supplementary file 6 — Reporting Summary [file 42003_2022_3638_MOESM6_ESM.pdf]

nature portfolio

COMMSBIO-22-0089C
Corresponding author(s): Jan Borgelt
Last updated by author(s): Jun 9, 2022

Reporting Summary

Nature Portfolio wishes to improve the reproducibility of the work that we publish. This form provides structure for consistency and transparency in reporting. For further information on Nature Portfolio policies, see our Editorial Policies and the Editorial Policy Checklist.

Statistics

For all statistical analyses, confirm that the following items are present in the figure legend, table legend, main text, or Methods section.

n/a	Confirmed	
☐	☒	The exact sample size (*n*) for each experimental group/condition, given as a discrete number and unit of measurement
☐	☒	A statement on whether measurements were taken from distinct samples or whether the same sample was measured repeatedly
☒	☐	The statistical test(s) used AND whether they are one- or two-sided<br>*Only common tests should be described solely by name; describe more complex techniques in the Methods section.*
☐	☒	A description of all covariates tested
☒	☐	A description of any assumptions or corrections, such as tests of normality and adjustment for multiple comparisons
☐	☒	A full description of the statistical parameters including central tendency (e.g. means) or other basic estimates (e.g. regression coefficient) AND variation (e.g. standard deviation) or associated estimates of uncertainty (e.g. confidence intervals)
☒	☐	For null hypothesis testing, the test statistic (e.g. *F*, *t*, *r*) with confidence intervals, effect sizes, degrees of freedom and *P* value noted<br>*Give P values as exact values whenever suitable.*
☒	☐	For Bayesian analysis, information on the choice of priors and Markov chain Monte Carlo settings
☒	☐	For hierarchical and complex designs, identification of the appropriate level for tests and full reporting of outcomes
☒	☐	Estimates of effect sizes (e.g. Cohen's *d*, Pearson's *r*), indicating how they were calculated

Our web collection on statistics for biologists contains articles on many of the points above.

Software and code

Policy information about availability of computer code

Data collection	All data handling was done using R version 4.0.3 in RStudio version 1.4.1103. Data were obtained from GBIF, OBIS and IUCN using the packages rgbif, robis, and rredlist. Handling of spatial and other data was conducted using the R packages caTools, doParallel, exactextractr, fasterize, maptools, parallel, raster, readxl, rgdal, rgeos, sf, sp, stringr, tidyverse, and xlsx, and in python using the arcpy module from ArcGIS Pro version 2.9.0. Code for preparing the data is available on GitHub (https://github.com/jannebor/dd_forecast).
Data analysis	Machine learning algorithms were trained and evaluated using the H2O.ai interface (Version 3.36.0.4) for R and the caret package. R code for training and testing the ML classifier, as well as applying the algorithm is available on GitHub (https://github.com/jannebor/dd_forecast). The classifier can be applied for single species using our web application (https://ml-extinctionrisk.indecol.no/).

For manuscripts utilizing custom algorithms or software that are central to the research but not yet described in published literature, software must be made available to editors and reviewers. We strongly encourage code deposition in a community repository (e.g. GitHub). See the Nature Portfolio guidelines for submitting code & software for further information.

Data

Policy information about availability of data

All manuscripts must include a data availability statement. This statement should provide the following information, where applicable:

- Accession codes, unique identifiers, or web links for publicly available datasets
- A description of any restrictions on data availability
- For clinical datasets or third party data, please ensure that the statement adheres to our policy

All data and code generated in this study is available without restrictions on GitHub (https://github.com/jannebor/dd_forecast).

Field-specific reporting

Please select the one below that is the best fit for your research. If you are not sure, read the appropriate sections before making your selection.

☐ Life sciences ☐ Behavioural & social sciences ☒ Ecological, evolutionary & environmental sciences

For a reference copy of the document with all sections, see nature.com/documents/nr-reporting-summary-flat.pdf

Ecological, evolutionary & environmental sciences study design

All studies must disclose on these points even when the disclosure is negative.

Study description	A machine learning classifier was developed to estimate threat rankings across Data Deficient species of the IUCN Red List of threatened species.
Research sample	All native, extant and not extinct species (n = 44,908) covered by the spatial dataset (only range maps) of the IUCN available from https://www.iucnredlist.org/resources/spatial-data-download.
Sampling strategy	Sample sizes were determined by amount of available data, i.e. number of species range maps available from https://www.iucnredlist.org/resources/spatial-data-download.
Data collection	The utilized collection consists of numerous open-access datasets (sources specified in the reference section).
Timing and spatial scale	Temporal alignment of the retrieved open-access datasets as good as possible, however, minor mismatches exist and are due to different dates of publication. Spatial scales were matched to a common resolution (30 arc minutes) by re-sampling.
Data exclusions	Species were excluded from the analysis if their assessed threat status was outdated according to IUCN. However, if fewer than five records remained for a given taxonomic class, outdated assessment were kept to ensure the taxonomic classes' inclusion in training the classifier.
Reproducibility	The code for reproducing the study is available on GitHub without restrictions (https://github.com/jannebor/dd_forecast).
Randomization	The data was randomly split into training and testing data using while maintaining original balances of environmental domains, taxonomic families and threat status.
Blinding	Blinding was not applicable for this study.

Did the study involve field work? ☐ Yes ☒ No

Reporting for specific materials, systems and methods

We require information from authors about some types of materials, experimental systems and methods used in many studies. Here, indicate whether each material, system or method listed is relevant to your study. If you are not sure if a list item applies to your research, read the appropriate section before selecting a response.

Materials & experimental systems

n/a	Involved in the study	
☒	☐	Antibodies
☒	☐	Eukaryotic cell lines
☒	☐	Palaeontology and archaeology
☒	☐	Animals and other organisms
☒	☐	Human research participants
☒	☐	Clinical data
☒	☐	Dual use research of concern

Methods

n/a	Involved in the study	
☒	☐	ChIP-seq
☒	☐	Flow cytometry
☒	☐	MRI-based neuroimaging
